# Directional and stabilizing selection shaped morphological, reproductive, and physiological traits of the invader *Solidago canadensis*

**DOI:** 10.1002/ece3.10410

**Published:** 2023-08-25

**Authors:** Leshan Du, Ayub M. O. Oduor, Wei Zuo, Haiyan Liu, Jun‐Min Li

**Affiliations:** ^1^ State Key Laboratory of Environmental Criteria and Risk Assessment Chinese Research Academy of Environmental Sciences Beijing China; ^2^ Zhejiang Provincial Key Laboratory of Plant Evolutionary Ecology and Conservation Taizhou University Taizhou China; ^3^ Department of Applied Biology Technical University of Kenya Nairobi Kenya; ^4^ Sanofi (Hangzhou) Pharmaceuticals Co. Ltd. Hangzhou China

**Keywords:** common garden, genetic differentiation, invasion ecology, local adaptation, natural selection, phenotypic differentiation

## Abstract

Trait evolution in invasive plant species is important because it can impact demographic parameters key to invasion success. Invasive plant species often show phenotypic clines along geographic and climatic gradients. However, the relative contributions of natural selection and neutral evolutionary processes to phenotypic trait variation among populations of invasive plants remain unclear. A common method to assess whether a trait has been shaped by natural selection or neutral evolutionary processes is to compare the geographical pattern for the trait of interest to the divergence in neutral genetic loci (i.e., *Q*_ST_ *–F*_ST_ comparisons). Subsequently, a redundancy analysis (RDA) can facilitate identification of putative agents of natural selection on the trait. Here, we employed both a *Q*_ST_ *–F*_ST_ comparisons approach and RDA to infer whether natural selection shaped traits of invasive populations of *Solidago canadensis* in China and identify the potential environmental drivers of natural selection. We addressed two questions: (1) Did natural selection drive phenotypic trait variation among *S. canadensis* populations? (2) Did climatic, latitudinal, longitudinal, and altitudinal gradients drive patterns of genetic variation among *S. canadensis* populations? We found significant directional selection for several morphological and reproductive traits (i.e., *Q*_ST_ *> F*_ST_) and stabilizing selection for physiological traits (i.e., *Q*_ST_ *< F*_ST_). The RDA showed that stem biomass of *S. canadensis* was strongly positively correlated with longitude, while leaf width ratio and specific leaf area were significantly positively correlated with the mean diurnal range. Stem biomass had a strong negative correlation with annual precipitation. Moreover, height of *S. canadensis* individuals was strongly positively correlated with altitude and precipitation of the wettest quarter. A longitudinal shift in precipitation seasonality likely selected for larger stem biomass in *S. canadensis*. Overall, these results suggest that longitudinal and altitudinal clines in climate exerted strong selection pressures that shaped the phenotypic traits of *S. canadensis*.

## Graphical abstract

There was significant directional selection for several morphological and reproductive traits and stabilizing selection for physiological traits. Populations at higher latitudes expressed larger mean values of morphological traits, while populations in higher altitudes expressed smaller mean values of the traits. Climatic conditions associated with latitudinal, longitudinal, and altitudinal clines likely exerted differential selection pressures on *Solidago canadensis* populations leading to genetic divergence among the populations.

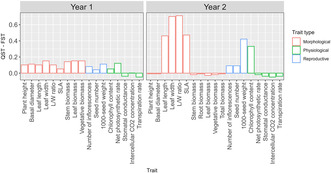

## 1 INTRODUCTION

Biological invasions by exotic plant species often reduce native biodiversity and disrupt ecosystem processes (Vila et al., 2011; Vitousek et al., 1997). Therefore, understanding the ecological and evolutionary processes that underlie phenotypic and genetic variation among invasive plant populations and the capacity of such populations to colonize a broad range of environments is a major goal in ecology. Phenotypic and genetic divergence among natural populations may result from natural selection processes and thus be adaptive (Linhart & Grant, 1996), or alternatively be caused by neutral (i.e., nonadaptive) evolutionary forces such as random colonization, spatially restricted gene flow, and genetic drift in peripheral populations (Campitelli & Stinchcombe, 2013; Endler, 1986). Populations can evolve adaptation to local environmental conditions if they possess sufficient heritable variation in phenotypic traits and natural selection is sufficiently strong (Hall & Willis, 2006). Indeed several invasive plant species have been shown to be locally adapted under a wide range of conditions (Oduor et al., 2016). However, the specific plant traits that are under natural selection and the traits that differ among populations due to neutral evolutionary forces have seldom been investigated (Exposito‐Alonso et al., 2018). Trait evolution in invasive plant species is important because it can impact demographic parameters key to invasion success (Hodgins et al., 2018).

Several invasive plant species have been shown to exhibit phenotypic clines along geographic gradients (Hodgins et al., 2018). For instance, clines in fecundity and growth‐related traits along geographic gradients have been reported in the exotic invaders *Lythrum salicaria* (Lythraceae) (Montague et al., 2008), *Impatiens glandulifera* (Balsaminaceae) (Kollman & Bañuelos, 2004), *Eschscholzia californica* (Papaveraceae) (Leger & Rice, 2007), *Ambrosia artemisiifolia* (Asteraceae) (van Boheemen et al., 2019), and *Alternanthera philoxeroides* (Amaranthaceae) (Yang et al., 2021). Because of the covariance between geography and many aspects of the abiotic (climatic and edaphic factors) and biotic (e.g., herbivory and competition) components of the environment, geographical gradients could select for genetically‐based intraspecific clines in quantitative traits across the introduced range (Broennimann et al., 2007). Intraspecific clines in life‐history traits with an underlying genetic basis might, therefore, indicate spatial variation in selection regimes leading to adaptive evolution (Alberto et al., 2013; Campitelli & Stinchcombe, 2013; Ledig et al., 2015; Sæther et al., 2007). Nevertheless, phenotypic clines may also result from neutral evolutionary processes (Soularue & Kremer, 2012; Vasemägi, 2006). Accordingly, phenotypic clines offer an opportunity to investigate the relative roles of adaptive and neutral evolutionary mechanisms affecting the geographical distribution of traits and allele frequencies in invasive species (Chun et al., 2011).

A common method to assess whether a phenotypic trait has been shaped by natural selection or neutral evolutionary processes is to compare the geographical pattern for the trait of interest to the divergence in putatively neutral genetic loci (Chun et al., 2009; Leinonen et al., 2008; Sun & Roderick, 2019; Xu et al., 2010). The level of genetic divergence in quantitative traits indicated by *Q*_ST_ may be influenced by natural selection, as well as by neutral evolutionary forces (Spitze, 1993). In contrast, the level of population divergence in neutral molecular markers indicated by *F*_ST_ is affected by neutral evolutionary forces and is often assumed to be selectively neutral (Wright, 1951). Thus, assuming no dominance or epistasis in a particular quantitative trait, comparisons of *F*_ST_ and *Q*_ST_ for any number of population pairs can yield one of three possible outcomes for the trait (Leinonen et al., 2008; Merilä & Crnokrak, 2001). First, if *Q*_ST_ of the trait is greater than *F*_ST_ (i.e., *Q*_ST_–*F*_ST_ > 0), then the trait is under directional selection for different local optima across populations. Second, if *Q*_ST_ = *F*_ST_ (i.e., *Q*_ST_–*F*_ST_ = 0), then the effects of natural selection and neutral evolutionary forces on the trait are not distinguishable and the trait is considered selectively neutral. Third, *Q*_ST_ < *F*_ST_ (i.e., *Q*_ST_–*F*_ST_ < 0) indicates that a trait is uniform across populations as a result of stabilizing selection (Leinonen et al., 2008, Merilä & Crnokrak, 2001). Ideally, *Q*_ST_ should be measured in a common garden setting to minimize or exclude differences in quantitative traits between plant populations that may arise from variation in ecological conditions between populations (Sun & Roderick, 2019).

The putative agents of natural selection causing adaptive differentiation among populations can be identified using ordination methods, including a redundancy analysis (RDA), which find significant associations between genetic polymorphism, phenotypic variation, and environmental variables (Capblancq et al., 2018; Capblancq & Forester, 2021). For instance, RDA revealed that partitioning of genetic variation in *Helichrysum italicum* (Asteraceae) was mainly associated with adaptation to temperature oscillations (Ninčević et al., 2021). In *Arabidopsis thaliana* (Brassicaceae), loci involved in adaptation to climate were identified using RDA (Lasky et al., 2012). The RDA can also be used to derive an adaptive index that predicts the performance of individuals in different environmental conditions (Capblancq et al., 2018). Therefore, RDA can complement *Q*_ST_ *–F*_ST_ comparisons in the detection of natural selection and the environmental variables driving natural selection.

Low or absence of genetic variation in phenotypic traits that result from founder events and genetic bottlenecks may constrain the capacity of invasive plants to respond evolutionarily to novel selection pressures in the exotic range (Sakai et al., 2001). However, multiple introduction events followed by genetic admixture of populations introduced from different sources in the native range (Dlugosch & Parker, 2008; Lavergne & Molofsky, 2007; Oduor et al., 2015; Rius & Darling, 2014) and/or single introduction events from highly genetically diverse native‐range source populations (Novak & Mack, 1993) may facilitate rapid adaptive evolution in invasive plants.

*Solidago canadensis* (Asteraceae) is a native of North America that is presently invasive in China. In China, the perennial forb was first introduced into the southeastern region for use in ornamental horticulture in 1935 and subsequently escaped to the wild (Dong et al., 2006; Du et al., 2017). Following escape from cultivation, it spread to colonize natural and agricultural ecosystems across southeastern China (Jin et al., 2020; Wan et al., 2020). A single mother plant produces numerous small wind‐dispersed seeds for long‐distance dispersal although the species also reproduces clonally (Dong et al., 2006). Previous studies found that *S. canadensis* exhibited latitudinal and longitudinal clines in quantitative traits in China (Du et al., 2017; Li et al., 2016, 2017). However, it remains unclear whether the clines in quantitative traits were an outcome of past natural selection or nonadaptive evolutionary forces.

Here, we employed both a *Q*_ST_ *–F*_ST_ comparisons approach and RDA to infer whether natural selection caused trait divergence among invasive populations of *S. canadensis* in China and identify the potential environmental drivers of natural selection. We addressed two questions: (1) Did natural selection drive phenotypic trait variation among *S. canadensis* populations? (2) Did climatic, latitudinal, longitudinal, and altitudinal gradients drive patterns of genetic variation among *S. canadensis* populations?

## 2 MATERIALS AND METHODS

### 2.1 Field sampling

**FIGURE 1:**
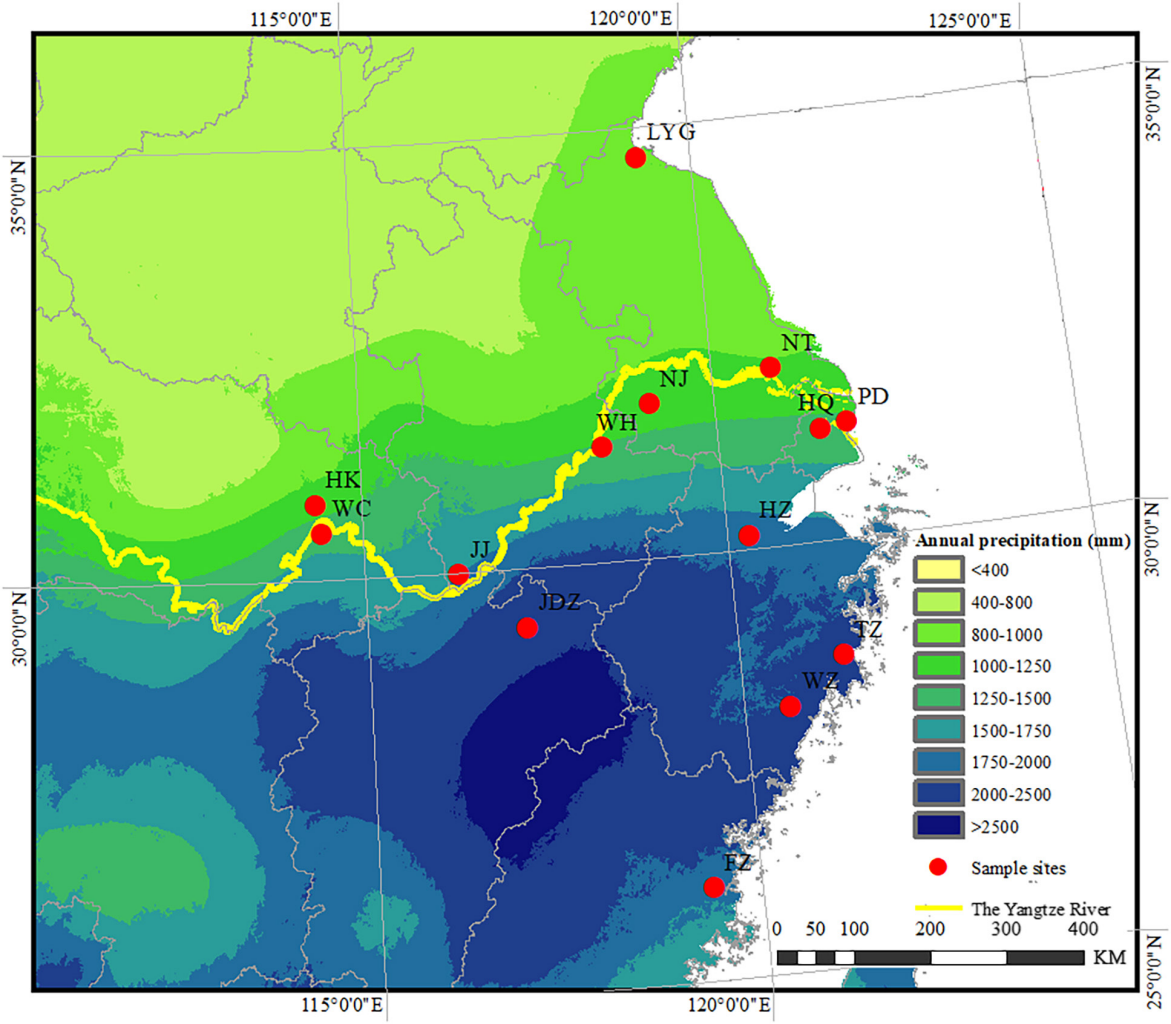
Map of 14 *Solidago canadensis* populations in China that were studied. The population abbreviations and details are presented in Table A1.

We sampled 14 *S. canadensis* populations (Figure 1; Table A1) in ruderal vegetation of China in October 2012. Twelve ramets that represented 12 families were selected in each of the 14 populations, for a total of 168 ramets. To minimize the chance of sampling individuals from the same maternal family more than once, within each population, we sampled ramets from sites that were separated from each other by at least 10 m. Shoots of the individual ramets were removed and the shoot bases with their attached rhizomes were dug up (see Li et al., 2016). The individual rhizomes were then wrapped in wet roll papers to keep moist and transferred to the laboratory until use in the common garden experiment as described below. To test for a geographical cline in phenotypic traits of *S. canadensis* among the 14 populations, we recorded the longitude, latitude, and altitude of the populations.

### 2.2 Pre‐experimental preparation of plantlets

To reduce environmental carry‐over effects, the field‐collected *S. canadensis* individuals were propagated vegetatively in a greenhouse for 4 months under uniform conditions. To achieve this, field‐sampled rhizomes were individually grown in plastic pots (30 cm × 30 cm; diameter × depth) that contained a mixture of field soil, sand, and peat in the ratio of 6:3:1, respectively. The field soil was collected from Linhai City, Zhejiang Province, China. The soil mixture had the following properties: pH 6.8, organic matter 27.66 g/kg, total nitrogen 361.0 mg/kg, available phosphorus 8.0 mg/kg, and available potassium 12.0 mg/kg.

### 2.3 Common garden experiment

In April 2013, we established a common garden experiment on Linhai campus of Taizhou University (121°17′ E, 28°87′ N) to assess quantitative trait variation among *S. canadensis* individuals from the 14 populations. We obtained three similar‐sized (ca. 15 cm) plantlets for each of the 12 families in the 14 populations from the pre‐experiment cultivation of plants described above. The plantlets were grown in a common garden in the field in three blocks. Thus, the total number of experimental plants was 504: 14 populations × 12 individuals (representing 12 maternal families) per population × 3 blocks. Within a block, the 12 individuals were planted in 12 separate plots that each measured 1.5 m × 1.5 m. The individual plants were grown 30 cm apart from each other. Throughout, the experimental plants received only rain‐fed water and were not fertilized.

### 2.4 Phenotypic trait measurement in the common garden

One month after transplant (21–27 May 2013), we took in situ measurements of photosynthesis on the third fully expanded leaf from the top using a portable photosynthesis meter (LI‐6400 XT, Li‐COR, Inc., Lincoln, NE, USA). The measurements were taken between 9:00 and 11:00 AM under the following conditions: a photosynthetically active radiation of 1400 μmol m^−2^ s^−1^, leaf temperature of 25°C, CO_2_ concentration of 400 ppm, and relative humidity of 70%. For each plant, we recorded net photosynthetic rate (*P*_n_), intercellular CO_2_ concentration (*C*_i_), stomatal conductance (*G*_s_), and transpiration rate (*T*_r_). These measurements were taken six times on different dates, and the average of the six rounds of measurements was used in the statistical analyses described below. Seven months after transplant (November 2013), we measured various morphological, physiological, and reproductive traits of the *S. canadensis* individuals. Individual plant height, leaf length (L), and leaf width (L) for the third leaf per plant were measured to a precision of 0.1 cm. We used the L and W dimensions to compute the L/W ratio. Chlorophyll content of the third leaf was measured using a portable chlorophyll meter. We also measured basal stem diameter to an accuracy of 0.01 cm. After 97% of the plants had flowered, we counted the total number of inflorescences per individual plant. Then after the plants had matured, we obtained a total seed count and 1000‐seed weight per plant. We also measured the total leaf area per plant using a WinFOLIA computer image analysis system (Regent Instruments Inc, Quebec, Canada) for use in the computation of specific leaf area (SLA). The above‐ground plant parts were then harvested and separated into shoots, leaves, and seeds. Biomass of the stem, leaf, and seed was measured after the samples had been dried at 80°C for 72 h. We measured biomass using an electronic balance to an accuracy of 0.01 g. To obtain the total vegetative biomass per *S. canadensis* individual, we summed up the oven‐dried stem and leaf biomass of the individuals. We computed SLA as the ratio of leaf area to leaf dry biomass.

A year later (November 2014), we repeated measurements of the same traits above from the same *S. canadensis* individuals that were not harvested. After the measurements had been taken, the whole root system for each *S. canadensis* individual was dug up and washed free of soil particles under running water. The roots and shoots were then dried at 80°C for 72 h and weighed individually to the nearest 0.01 g.

### 2.5 Analysis of neutral genetic diversity

#### 2.5.1 Leaf sampling

In October 2012, we collected leaf tissues from 30 *S. canadensis* individuals per population in the same 14 populations that we sampled for rhizomes as described above (Figure 1; Table A1). The sampled leaves were immediately immersed in self‐sealing plastic bags that contained silica gel and then stored in the laboratory at room temperature until use in DNA extraction.

### 2.6 Microsatellite analysis

In November 2012, genomic DNA was extracted from 0.1 g of each leaf sample using a modified sodium dodecyl sulfate protocol on a FastPrep‐24 Automated Lysis and Homogenization System (MP Biomedicals, Santa Ana, CA, USA). Total DNA concentration was determined with a NanoDrop 2000 Lite Spectrophotometers (ThermoFisher Scientific, Inc. Rockford, IL, USA). The DNA samples were then diluted to 10 ng/μL and stored at −20°C until use in simple sequence repeats (SSR) analyses. Five primer pairs (synthesized by Boshang Biotechonology Co., Ltd. in China) were used in SSR amplifications (Table A2) (Wieczorek & Geber, 2002). We ran PCR in a 20 μL volume that was made up of 1 × PCR reaction buffer, 1.5 mM Mg^2+^, 4 ng template DNA, 0.2 μM each of forward and reverse primers, 0.2 mM 4 × dNTP mixture, and 1 U Taq polymerase (Promega Cooperation, Madison, WI, USA). We performed PCR amplifications using a PTC 220 Peltier Thermal Cycler (Bio‐Rad Laboratories, Hercules, CA, USA) with the following settings: denaturation at 95°C for 5 min, followed by 34 cycles of 30 s at 95°C, 30 s at 58°C (−1°C per cycle), 45 s at 72°C, with a final elongation of 7 min at 72°C. We analyzed the PCR products using a Fragment Analyzer™ Automated CE System (Advanced Analytical Technologies, Inc, Ankeny, IA, USA) with an 80 cm‐long capillary column. We used a DNF‐900 35–500 bp ds DNA Reagent Kit in the analysis. We genotyped DNA fragments using PROSize® 2.0 Data Analysis Software based on the elution time compared with a size standard.

### 2.7 Statistical analyses

#### 2.7.1 A test for phenotypic trait variation among the *S. canadensis* populations

We ran general linear models to test whether the different *S. canadensis* populations exhibited significant variation in phenotypic trait expressions in the common garden experiment. In the models, identity of *S. canadensis* population was included as a fixed term, while family and plot were treated as random terms. We specified morphological, physiological, and reproductive traits of *S. canadensis* individuals as response variables. Statistical significance of the factors was tested using *F*‐statistic. The models were run separately for measurements that we took in year 1 and year 2 of the experiment to assess whether the plant traits would be expressed consistently during the lifetime of *S. canadensis*. The statistical analyses were performed in SPSS v. 19.0.

#### 2.7.2 Analysis of molecular data

We used POPGENE v. 1.31 to compute the number of loci (*N*), percentage of polymorphic loci (*P* %), number of different alleles (*N*_a_), number of effective alleles (*N*_e_), Nei's gene diversity per locus (*H*_S_), and Shannon's information index (*I*). We performed an analysis of molecular variance (AMOVA) (Excoffier et al., 1992) to assess how genetic variation was distributed among and within the populations. The AMOVA was performed in GenAlEx v. 6.501 (Peakall & Smouse, 2005). The significance of genetic differentiation was tested using *F*‐statistic with 1000 random permutations.

#### 2.7.3 Comparisons between *Q*_ST_ and *F*_ST_

We compared quantitative genetic (*Q*_ST_) and neutral molecular marker (*F*_ST_) differentiation among the 14 populations to infer the relative contributions of natural selection and random genetic drift to clinal variation in quantitative traits of *S. canadensis*. We computed the among‐population coefficient of gene differentiation (*F*_st_) based on microsatellite DNA using GenAlEx v. 6.501 (Peakall & Smouse, 2005). We computed the *Q*_ST_ index based on the physiological, growth, and reproductive traits of the different *S. canadensis* populations by applying the formula *Q*_ST_ = *V*_pop_/(*V*_pop_ + 2*V*_ind_), wherein *V*_pop_ and *V*_ind_ represent variation in the quantitative traits among and within populations, respectively (Spitze, 1993). We then computed the difference between the resultant *Q*_ST_ value for each trait and the common *F*_ST_ of 0.078 (i.e., *Q*_ST_ – 0.078) (see results below on population genetic structure) to infer the effects of natural selection vs random genetic drift.

#### 2.7.4 Test for associations between phenotypic variation and environmental variables

To test whether latitude, longitude, altitude, and 19 bioclimatic variables were significantly correlated with variation in quantitative traits among the *S. canadensis* populations, we performed an RDA. The 19 bioclimatic variables (BIO1 = Annual Mean Temperature, BIO2 = Mean Diurnal Range (Mean of monthly (max temp – min temp)), BIO3 = Isothermality (BIO2/BIO7) (×100), BIO4 = Temperature Seasonality (standard deviation ×100), BIO5 = Max Temperature of Warmest Month, BIO6 = Min Temperature of Coldest Month, BIO7 = Temperature Annual Range (BIO5‐BIO6), BIO8 = Mean Temperature of Wettest Quarter, BIO9 = Mean Temperature of Driest Quarter, BIO10 = Mean Temperature of Warmest Quarter, BIO11 = Mean Temperature of Coldest Quarter, BIO12 = Annual Precipitation, BIO13 = Precipitation of Wettest Month, BIO14 = Precipitation of Driest Month, BIO15 = Precipitation Seasonality (Coefficient of Variation), BIO16 = Precipitation of Wettest Quarter, BIO17 = Precipitation of Driest Quarter, BIO18 = Precipitation of Warmest Quarter, and BIO19 = Precipitation of Coldest Quarter) were downloaded from the WorldClim database (http://www.world‐clim.org/current) using DIVA‐GIS v. 7.2.1.1. The 19 bioclimatic variables were based on historical climate data for the years 1950–2000, which were interpolated at 30 arc‐seconds resolution (ca. 1 km^2^ resolution) (Hijmans et al., 2005). A redundancy analysis is an expansion of multiple linear regression, in that multiple explanatory variables are used to explain multiple response variables (Legendre & Legendre, 2012). Collinear variables (BIO1, BIO4–BIO11, BIO13–BIO17, BIO18 & BIO19) were removed to minimize collinearity, as assessed with variable inflation factor (VIF max <10). Moreover, we did not use molecular data in the RDA to test for associations between genetic polymorphism, phenotypic variation, and environmental variables because the VIF factor for all the five loci was greater than the threshold value of 10. Thus, altitude, latitude, longitude, BIO2, BIO3, BIO12, and BIO16 were retained in the subsequent RDA. We applied forward selection to the data using the *ordiR2step* vegan function with the following stopping criteria: variable significance of *p* < .01 using 1000 permutations, and the adjusted *R* ^2^ of the global model. We standardized the predictors (i.e., subtracted the mean and divided by the standard deviation) to ensure that the variable units were comparable (Legendre & Legendre, 2012). Tests of significance of the global RDA model (containing all the significant variables), individual RDA axes, and individual explanatory variables were performed using a permutational analysis of variance with 999 permutations. The RDA was performed using the Vegan package v. 2.6‐4 (Oksanen et al., 2022) in R v. 4.2.3 (R Core Team, 2023).

## 3 RESULTS

### 3.1 Phenotypic trait variation among *S. canadensis* populations

In the first and second years of the experiment, the *S. canadensis* individuals exhibited a significant among‐population variation in the growth and reproductive traits (Table A3). However, the *S. canadensis* individuals did not show significant among‐population variation in physiological traits except for relative chlorophyll content (Table A3). Moreover, height and stem diameter of the *S. canadensis* individuals did not vary among the populations in the second year (Table A3)

### 3.2 Genetic diversity and structure in *S. canadensis* populations

We scored a total of 49 bands from the five SSR primers. The populations exhibited variability in the genetic diversity indices *P*, *N*a, *N*e, *H*, and *I* (Table 1). The highest genetic diversity was found in the WH population (*P* = 100%, *N*_a_ = 2.000, *N*_e_ = 1.347, *H*_S_ = 0.242, and *I* = 0.395), while the lowest genetic diversity (*P* = 91.84%, *N*_a_ = 1.918, *N*_e_ = 1.296, *H*_S_ = 0.205, and *I* = 0.340) occurred within the WZ population (Table 1). The AMOVA revealed that most of the genetic variation occurred within the *S. canadensis* populations (92.186%) (Table 2). There was low genetic differentiation among the *S. canadensis* populations (*F*_ST_ = 0.078). Estimated gene flow (*N*_m_) among the populations was 2.955.

**TABLE 1:** Genetic diversity indices of *Solidago canadensis* at the population and species level.

Population	Number	*N*	*P* (%)	*N*a	*N*e	*H*_S_	*I*
FZ	30	45	91.84	1.918	1.316	0.219	0.358
HK	30	47	95.92	1.959	1.337	0.2333	0.382
HZ	30	47	95.92	1.959	1.317	0.219	0.362
HQ	30	49	100.00	2.000	1.332	0.229	0.378
JDZ	30	48	97.96	1.979	1.349	0.240	0.389
JJ	30	46	93.88	1.939	1.338	0.229	0.372
LYG	30	47	95.92	1.959	1.307	0.216	0.357
NJ	30	48	97.96	1.979	1.313	0.225	0.376
NT	30	45	91.84	1.918	1.339	0.228	0.369
PD	30	45	91.84	1.918	1.341	0.228	0.369
TZ	30	48	97.96	1.979	1.329	0.229	0.378
WZ	30	45	91.84	1.918	1.296	0.205	0.340
WC	30	48	97.96	1.979	1.328	0.226	0.372
WH	30	49	100.00	2.000	1.347	0.242	0.395
Population mean (SD)	30	47	95.77 (3.04)	1.958 (0.03)	1.328 (0.05)	0.226 (0.01)	0.371 (0.01)
Species mean (SD)	420	49	100.00	2.00 (0.00)	1.335 (0.143)	0.243 (0.079)	0.402 (0.103)

*Note*: *N* is the number of polymorphic loci; *P* is the percentage of polymorphic loci; *N*a is the observed number of alleles; *N*e is the effective number of alleles; *H*_S_ is Nei's gene diversity per locus; *I* is the Shannon index. SD indicates standard deviation.

**TABLE 2:** Results of analysis of molecular variance (AMOVA) that tested for genetic variation among and within 14 populations of *Solidago canadensis*.

Source of variation	*d.f*.	*SS*	*MS*	Variance components	Percentage of variation (%)	*p*
Among populations.	13	385.712	29.670	0.710	7.814	<.001
Within populations.	406	3399.967	8.374	8.374	92.186	<.001
Total	419	3785.679	38.044	9.084		

Abbreviations: *d.f*., degree of freedom; SS, sum of squares; MS, expected mean squares.

### 3.3 Phenotypic versus genetic differentiation

**FIGURE 2:**
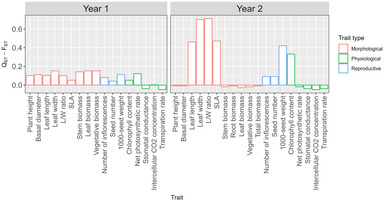
Comparisons of quantitative and genetic differentiation (*Q*_ST_–*F*_ST_ values) among *Solidago canadensis* individuals from 14 populations that were grown in a common garden experiment over 2 years (Year 1 & Year 2).

In the first year of the experiment, *Q*_ST_ values of all the morphological and reproductive traits and two physiological traits (i.e., chlorophyll content and net photosynthetic rate) were larger than the common *F*_ST_ value of 0.078 (i.e., *Q*_ST_–*F*_ST_ > 0; Figure 2). However, *Q*_ST_ values of the physiological traits stomatal conductance, intercellular CO_2_ concentration, and transpiration rate were lower than the common *F*_ST_ value (i.e., *Q*_ST_–*F*_ST_ < 0; Figure 2). In the second year of the experiment, *Q*_ST_ values of leaf length, leaf width, L/W ratio, SLA, number of inflorescences, seed number, 1000‐seed weight, and relative chlorophyll content were larger than the common *F*_ST_ value (i.e., *Q*_ST_–*F*_ST_ > 0), while *Q*_ST_ values of the other quantitative traits were lower than *F*_ST_ (i.e., *Q*_ST_–*F*_ST_ < 0; Figure 2).

### 3.4 Environmental predictors of phenotypic trait variation in S. canadensis populations

**FIGURE 3:**
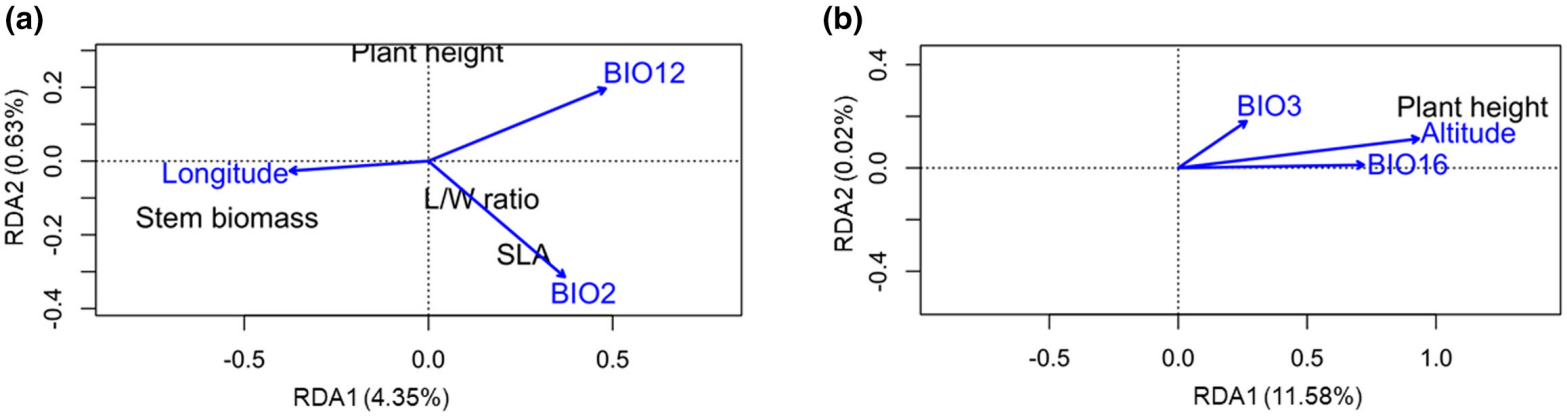
Redundancy analysis (RDA) ordinate plots showing relationships between longitude, altitude, and the bioclimatic variables BIO2, BIO3, BIO12, and BIO16 and traits (stem biomass, leaf/width (L/W) ratio, specific leaf area (SLA), and height) of *Solidago canadensis* individuals from 14 populations that were measured in year 1 (a) and year 2 (b) in a common garden experiment. Environmental variables are represented by blue arrows. Length of arrows represents the relative importance of that variable. Loadings of *S. canadensis* traits on the RDA axes showing the direction of correlations (positive or negative) between the traits and environmental variables are shown in Appendices A4 and A5.

In the RDA for traits that were measured in the first year of the experiment, the global RDA model was significant (*F* = 7.60; *p* = .001) and each of the three environmental variables (longitude, BIO2, and BIO12) that were included in the model were significant (*p <* .05). The three environmental variables explained 4.5% of the variation in phenotypic traits of *S. canadensis*. The first axis (RDA 1) was significant (*F* = 18.66; *p* = .001) and primarily associated with annual precipitation (BIO12), while longitude and mean diurnal range (BIO2) similarly drove variation along the axis but in the opposite directions (Table 3). The second axis (RDA 2) was only marginally significant (*F* = 4.14; *p* = .083) and driven by the mean diurnal range (BIO2) and annual precipitation (BIO12) (Table 3). The third axis was not significant (*F* = 0.016; *p* = .998). The stem biomass of *S. canadensis* was positively correlated with longitude, while the L/W ratio and SLA were positively correlated with mean diurnal range (BIO2) (Figure 3a). However, stem biomass had a negative correlation with annual precipitation (BIO12) (Figure 3a). The three traits had relatively higher loadings on RDA 1 compared to the other traits (Table A4). For the *S. canadensis* traits that were measured in the second year of the experiment, the overall model was significant (*F* = 18.18; *p* = .001) and each of the three environmental variables, including altitude, BIO3, and BIO16, that were included in the model was significant (*p <* .05). The three environmental variables explained 10.9% of the variation in phenotypic traits of *S. canadensis*. The first axis (RDA 1) was significant (*F* = 54.48; *p* = .001) and primarily driven by altitude and precipitation of the wettest quarter (BIO16) (Table 4). Isothermality (BIO3) had a relatively weak correlation (0.24) with RDA 1 (Table 4). Height of *S. canadensis* at maturity was positively correlated with altitude and precipitation of the wettest quarter (BIO16) (Figure 3b). Plant height had a relatively higher loading on RDA 1 compared to the other traits (Table A5). The third axis was not significant (*F* = 0.002; *p* = 1.00) (Table 4). However, none of the physiological traits of *S. canadensis* were significantly correlated with environmental variables (results not shown).

**TABLE 3:** Results of a redundancy analysis that tested for correlations between the environmental variables latitude, longitude, altitude, and 19 bioclimatic variables and phenotypic traits of *Solidago canadensis* from 14 populations that were measured in the first year of the experiment.

	RDA1	RDA2	RDA3
*P* (Permutational test of significance)	0.001	0.083	0.998
Eigenvalue	0.0003	0.00007	0.0000003
% Variance explained	81.0	18.1	0.01
Cumulative % variance explained	81.0	99.9	100.0
Constraining variable contributions			
Longitude	−0.58	−0.061	0.80
Mean diurnal range (BIO2)	0.57	−0.71	−0.38
Annual precipitation (BIO12)	0.75	0.44	0.48

**TABLE 4:** Results of a redundancy analysis that tested for correlations between the environmental variables latitude, longitude, altitude, and 19 bioclimatic variables and phenotypic traits of *Solidago canadensis* from 14 populations that were measured in the second year of the experiment.

	RDA1	RDA2	RDA3
*P* (Permutational test of significance)	0.001	0.995	1.000
Eigenvalue	0.0008	0.0000011	0.00000003
% Variance explained	99.85	0.15	0.00
Cumulative % variance explained	99.85	100.00	100.00
Constraining variable contributions			
Altitude	0.85	0.53	−0.029
Isothermality (BIO3)	0.24	0.85	0.468
Precipitation of wettest quarter (BIO16)	0.65	0.06	0.76

## 4 DISCUSSION

Population differentiation in growth and reproductive traits as well as chlorophyll content was evident in our range‐wide comparison of the invader *S. canadensis* in China (Table A3). Because *S. canadensis* individuals from the various populations were grown under uniform conditions in a common garden, intraspecific variation is likely due to genetic variation in the traits. The *Q*_ST_ *–F*_ST_ comparisons found evidence of directional selection for morphological and reproductive traits and stabilizing selection for physiological traits (Figure 2), while the RDA found significant associations between phenotypic variation and environmental variables (Figure 3). Because clines in phenotypic traits with an underlying genetic basis potentially indicate natural selection (Alberto et al., 2013; Campitelli & Stinchcombe, 2013), it is plausible that natural selection is the principal force that shaped the genetic architecture of *S. canadensis* populations.

### 4.1 *Q*_ST_–*F*_ST_ comparisons suggest that directional and stabilizing selection shaped phenotypic traits of invasive populations of *S. canadensis*

For all the reproductive traits and some morphological and physiological traits, *Q*_ST_ values were greater than *F*_ST_ values in both years of the experiment (i.e., *Q*_ST_–*F*_ST_ > 0) (Figure 2), which suggests that directional natural selection, rather than neutral evolutionary processes, favored those traits. In contrast, for some physiological traits, *Q*_ST_ values were lower than *F*_ST_ values (i.e., *Q*_ST_ *–F*_ST_ < 0) in both years of the experiment (Figure 2), which indicates that mean values of those traits were shaped by stabilizing selection. Molecular phylogeography data suggest that populations of *S. canadensis* in China were founded through multiple introduction events from the North American native range (Wan et al., 2020; Zhao et al., 2015). The multiple introduction events likely caused high‐standing genetic diversity within the introduced *S. canadensis* populations, which then enabled them to respond evolutionarily to selection imposed by the novel environmental conditions. Moreover, *S. canadensis* has undergone demographic and range expansion in China (Wan et al., 2020). Demographic and range expansions can cause an accumulation of rare alleles and low‐frequency mutations (Fu, 1997; Tajima, 1989). As *S. canadensis* has invaded China for ca. 80 years, which is equivalent to several overlapping generations, it is also likely that over time, demographic and range expansions increased standing genetic diversity that natural selection acted upon.

One striking result in the present study was low genetic differentiation (*F*_ST_ = 0.078) among *S. canadensis* populations despite divergence in growth and reproductive traits among the populations. A possible explanation for this finding is that there was a high degree of gene flow, which led to admixed populations. *Solidago canadensis* reproduces both clonally and sexually (Li et al., 2017). It is likely that gene flow occurred among widely spaced *S. canadensis* populations through long‐distance pollen flow and seed dispersal (the estimated gene flow was high at 2.955). Studies on other plant species have found evidence of diversifying selection and local adaptation in spite of strong gene flow among populations of those species (Chun et al., 2011; Sæther et al., 2007; Sanou et al., 2005), which may be an outcome of positive covariance of allele frequencies among populations (Latta, 2003). It is also possible that the relatively weak population genetic structure that we detected based on neutral genetic markers in the present study may not be strongly indicative of population differentiation in adaptive genes and quantitative traits (sensu Leinonen et al., 2008). Microsatellites have been used to obtain an unbiased estimate of genomic diversity based on the assumption that they are selectively neutral (Väli et al., 2008). However, microsatellites can grossly underestimate significant differences in genetic diversity and structure among populations (Väli et al., 2008). Therefore, future studies may employ high‐throughput DNA sequencing techniques that can identify and score genetic markers that are randomly distributed across the entire genome to more vigorously assess genetic diversity and structure among the invasive populations of *S. canadensis*.

The *Q*_ST_ *–F*_ST_ values for some morphological and physiological traits of *S. canadensis* were positive in the first year but negative in the second year of the experiment (Figure 2), which suggests that those traits displayed phenotypic plasticity. Between‐year variation in phenotypes may be an adaptation strategy to changing environmental conditions (Ledig et al., 2015). Although we did not measure environmental variables in the experimental site over the 2 years of study, it is likely that there was between‐year variation in climatic elements and soil conditions at the study site to which some traits of *S. canadensis* displayed plastic responses. Phenotypic plasticity is thought to play an important role in biological invasions by allowing species to survive and reproduce under a wide range of environmental conditions (Richards et al., 2006). For some exotic plant species, both local adaptation and phenotypic plasticity may jointly facilitate invasion. For instance, both local adaptation and phenotypic plasticity were linked to successful invasion by *Wedelia trilobata* (Asteraceae) (Si et al., 2014), *A. artemisiifolia* (Xiong et al., 2023), and *Prunella vulgaris* (Lamiaceae) (Godoy et al., 2010). It is likely that phenotypic plasticity and local adaptation both underlie the invasiveness of *S. canadensis*.

Similar to the present findings, other studies found that natural selection‐shaped traits of invasive plant species. For instance, diversifying selection caused divergence in reproductive traits among invasive populations of *A. artemisiifolia* (Chun et al., 2011). In *L. salicaria*, time to first flower and size at reproduction were influenced by stabilizing selection (Colautti & Barrett, 2010). Directional selection for flowering time caused adaptive evolution in the invader of agro‐ecosystems *Raphanus raphanistrum* (Brassicaceae) (Ashworth et al., 2016). These findings broadly support the idea that contemporary evolution of local adaptation can determine invasive species' success (Hodgins et al., 2018).

### 4.2 Putative agents of natural selection inferred from a redundancy analysis

**FIGURE A1:**
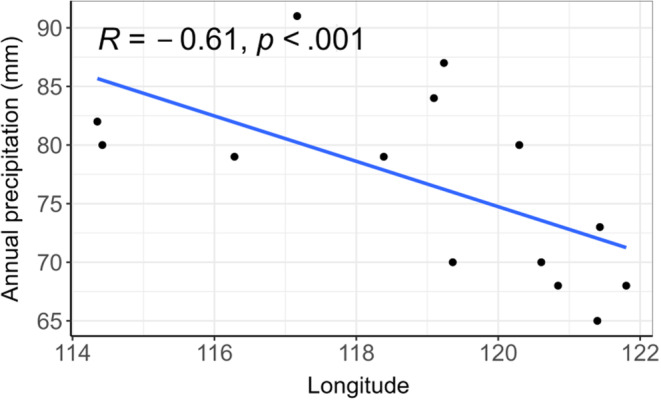
Results of a linear regression showing a longitudinal cline in historical mean annual precipitation (BIO12) in China over the period 1950–2000.

The results of a RDA showing a positive correlation between stem biomass of *S. canadensis* and longitude and a negative association between stem biomass and annual precipitation (BIO12) (Figure 3a) indicate that longitudinal cline in precipitation pattern selected for variable stem biomass. Generally, precipitation varies considerably with changes in longitude in many parts of the world, including China (Wang et al., 2020). We found that mean historical annual precipitation (BIO2) for the period 1950–2000 had a negative linear relationship with longitude in the study area (Figure A1). Therefore, it is likely that the relatively drier soils at higher longitudes selected for *S. canadensis* genotypes with larger stems. A previous study found that *S. canadensis* developed larger roots under drought conditions (Du et al., 2017), likely as a strategy to extract more water from a drier soil. Another study found a longitudinal cline in relative biomass allocation to roots and shoots and efficiency in water utilization in *S. canadensis* (Li et al., 2016). Similarly, *Capparis spinosa* (Capparaceae) developed larger stems when grown under drought conditions (Gan et al., 2013). Thus, *S. canadensis* individuals with larger stems may become adapted and more successful invaders at higher longitudes where there is reduced precipitation. Such individuals may also have stronger negative ecological impacts given that size of invasive plants can be positively associated with their impacts (Parker et al., 1999; Sun et al., 2013).

The present finding of a longitudinal cline in stem biomass is in accord with those of other studies that observed longitudinal clines in plant phenotypes and phenology (Wang et al., 2020). For instance, the mean leaf size and the internode length of *Cynodon dactylon* (Poaceae) had a significant negative relationship with longitude (Wang et al., 2020). East‐to‐west longitudinal genetic clines in morphological traits and tolerance against environmental conditions were observed in *Abies sachalinensis* (Pinaceae) (Kitamura et al., 2020). In another study, North American populations of *A. thaliana* showed longitudinal clines in flowering time (Samis et al., 2012). Overall, these studies suggest that different combinations of environmental factors along a longitudinal gradient may impose variable selection pressures on plants leading to longitudinal differentiation among plant populations.

That the leaf traits L/W ratio and SLA were significantly positively correlated with the mean diurnal range (BIO2) (Figure 3a) supports the suggestion that spatial variation in SLA often reflects plant responses to variation in temperature (Rosbakh et al., 2015). It is generally accepted that SLA is correlated with the temperature conditions of a habitat (Poorter et al., 2009). In particular, plant species with low SLA will mainly be found in the colder part of a temperature gradient, whereas species with relatively high SLA values should be largely restricted to sites exposed to higher temperatures (Poorter et al., 2009). Due to the positive linear relationship that SLA has with the relative growth rate of plants (Poorter et al., 2009), it is plausible that *S. canadensis* populations that occur in warmer regions will have higher SLA and consequently higher relative growth rates and fecundity than *S. canadensis* populations that occur in colder regions.

Our results showing that height of *S. canadensis* at maturity was strongly positively correlated with precipitation of the wettest quarter (BIO16) (Figure 3b) are in agreement with those of a global synthesis, which found that plant height was positively correlated with precipitation in the wettest month (Moles et al., 2009). These results suggest that precipitation in the wettest quarter might be a strong selective agent on *S. canadensis* height. However, the present finding that height of *S. canadensis* at maturity was positively correlated with altitude (Figure 3b) contradicts results of the global synthesis, which found that altitude was a poor predictor of plant height (Moles et al., 2009). As plant height can influence plant competitive ability above‐ground (Gioria & Osborne, 2014), the present results suggest that *S. canadensis* populations that occur at higher altitudes may have faced strong above‐ground competition from the resident flora, which may have led to the evolution of taller stature in *S. canadensis*.

### 4.3 Perspectives for future study

The RDA models explained only 4.5% and 11.6% of the variation in phenotypic traits in the first and second year of the experiment, respectively, which indicates that the environmental variables that were included in the models were not the only likely forces of natural selection in the populations under study. It is possible that other environmental variables that we did not measure also imposed selection on *S. canadensis* traits. Apart from climatic factors, soil biota can drive natural selection on plant traits (Lau & Lennon, 2011). A recent study suggests that older populations of *S. canadensis* interact with more beneficial and fewer pathogenic soil microorganisms than younger populations of *S. canadensis* in China (Oduor et al., 2022). Hence, future studies may explore whether differences in microbial community structure influence selection on *S. canadensis* traits.

Assessments of geographic clines in the traits of invasive plant populations are often used to infer adaptive evolution in invasive plants although this approach may sometimes overestimate the full extent of local adaptation (Frenne et al., 2013). Geographical clines can in principle also be explained by neutral evolutionary processes (Endler, 1977). Moreover, separate introductions from native populations to similar biogeographic regions in the introduced range can result in parallel clines in the absence of selection. Therefore, approaches that include careful sampling with respect to environmental gradients, genome‐wide analysis of neutral genetic markers, and reciprocal transplants in multiple locations among different climatic regions may be employed in future studies to more rigorously test for adaptive genetic variation in fitness‐related traits among *S. canadensis* populations.

## 5 CONCLUSION

Our complementary use of *Q*_ST_ *–F*_ST_ comparisons and RDA revealed that both directional selection and stabilizing selection shaped phenotypic traits of *S. canadensis* populations, and that climatic factors, mean diurnal range (BIO2), annual precipitation (BIO12), and precipitation of the wettest quarter (BIO16),acted as dynamic selective agents that promoted diversification of morphological and reproductive traits across the populations. Future field experiments evaluating the role of biotic interactions and ecophysiological function in a wide range of climatic conditions will be necessary to confirm the selective agents that produced the clines in phenotypic traits observed here.

## AUTHOR CONTRIBUTIONS

**Leshan Du:** Data curation (equal); formal analysis (equal); investigation (equal); methodology (equal); writing – original draft (equal). **Ayub M.O. Oduor:** Conceptualization (supporting); methodology (equal); writing – original draft (equal); writing – review and editing (equal). **Wei Zuo:** Data curation (equal); formal analysis (equal); investigation (equal); methodology (equal). **Haiyan Liu:** Data curation (equal); formal analysis (equal); investigation (equal); methodology (equal). **Junmin Li:** Conceptualization (lead); data curation (equal); formal analysis (equal); funding acquisition (lead); investigation (equal); methodology (equal); project administration (lead); resources (lead); writing – original draft (equal); writing – review and editing (equal).

## FUNDING INFORMATION

This work was supported financially by the National Natural Science Foundation of China (No. 31850410484 & No. 31270461), the Ten Thousand Talent Program of Zhejiang Province (2019R52043), the National Key Research and Development Program of China (2016YFC1201100), and Taizhou city 500 Talent Program.

## Data Availability

The datasets used in the study are publicly accessible through Dryad digital repository (https://doi.org/10.5061/dryad.9w0vt4bn0).

## APPENDIX A

**TABLE A1:** Sample locations, climatic and geographical information of 14 *Solidago canadensis* populations in China.

No.	Population	Location	Longitude	Latitude	Altitude	Annual mean temperature (°C)	Temperature seasonality (standard deviation)	Annual precipitation (mm)	Precipitation seasonality (coefficient of variation)
1	FZ	Fuzhou City, Fujian Province	119.359° E	26.098° N	19	20.1	64.96	1375	51
2	HZ	Hangzhou City, Zhejiang Province	120.297° E	30.161° N	9	16.9	85.09	1356	42
3	LYG	Lianyungang City, Jiangsu Province	119.235° E	34.654° N	3	13.5	94.59	864	88
4	NT	Nantong City, Jiangsu Province	120.843° E	32.07° N	5	15.0	85.38	1043	54
5	MH	Minhang District, Shanghai City	121.433° E	31.307° N	5	16.2	83.65	1068	47
6	PD	Pudong District, Shanghai Province	121.804° E	31.354° N	3	15.9	81.46	1022	46
7	TZ	Taizhou City, Zhejiang Province	121.397° E	28.656° N	6	17.5	75.19	1571	47
8	WZ	Wenzhou City, Zhejiang Province	120.607° E	28.126° N	4	16.0	73.36	1774	49
9	HK	Hankou District, Wuhan City, Hubei Province	114.350° E	30.878° N	25	17.1	89.46	1190	55
10	WC	Wuchang District, Wuhan City, Hubei Province	114.421° E	30.541° N	26	17.3	89.34	1252	57
11	JJ	Jiujiang City, Jiangxi Province	116.283° E	29.985° N	18	17.2	87.42	1420	49
12	JDZ	Jingdezhen City, Jiangxi Province	117.166° E	29.318° N	40	17.7	83.07	1717	55
13	WH	Wuhu City, Anhui Province	118.387° E	31.342° N	16	16.3	91.65	1175	49
14	NJ	Nanjing City, Jiangsu Province	119.094° E	31.794° N	22	15.7	90.16	1075	51

**TABLE A2:** Simple sequence repeat (SSR) primer sequences that were used in the present study.

Primer	Sequence (5′–3′)	Tm (°C)	Motif	Fragment size (bp)
SS1B	F: TTCCTGAAGAAGCTTCGCATA R: CAGCAGCATGCATTCCATAA	61	(GTA)8	156–210
SS4F	F: ACACGTGGACCAGGTAAAGC R: CGCGAAGAACAGCAATACAA	51	(CTT)7	168–192
SS4G	F: TGTGACAGCTTGTTAACTTTATACTGA R: CACCCCCTTTCCAAATATGA	48	(CT)10	171–227
SS20E	F: CACACAGACACTCAAAGCTTCA R: ACCCGCCCTAAAAATAAAGA	51	(TA)4(TG)12	273–299
SS24F	F: AGVTTTTCTTCGCCATTTCCTTCC R: AATTTGGTTACTGGGTTTTCTTGA	53	(CAT)8	156–222

**TABLE A3:** Results of general linear models that tested the effects of *Solidago canadensis* population and family identities on phenotypic trait expression by *S. canadensis* individuals that were grown in a common garden over 2 years (Year‐1 and Year‐2).

Phenotypic traits																		
	Year‐1									Year‐2								
	Population			Family			Plot			Population			Family			Plot		
	*d.f*	*F*	*p*	*d.f*	*F*	*p*	*d.f*	*F*	*p*	*d.f*	*F*	*p*	*d.f*	*F*	*p*	*d.f*	*F*	*p*
Morphological traits																		
Plant height	13	4.91	<.01	135	2.57	<.01	24	0.72	.83	13	1.35	.19	136	1.19	.12	24	11.72	<.01
Basal diameter	13	6.09	<.01	135	1.44	.01	24	1.04	.42	13	0.93	.52	136	0.98	.54	24	2.95	<.01
Leaf length	13	2.36	.01	135	2.23	<.01	24	0.82	.71	13	5.05	<.01	28					
Leaf width	13	7.96	<.01	135	3.00	<.01	24	1.28	.18	13	15.13	<.01	28					
L/W ratio	13	5.82	<.01	135	4.39	<.01	24	0.73	.82	13	16.21	<.01	28					
SLA	13	4.14	<.01	135	1.78	<.01	24	1.28	.18	13	5.36	<.01	28					
Stem biomass	13	5.97	<.01	135	1.64	<.01	24	0.90	.60	13	1.68	.07	136	1.21	.10	24	4.38	<.01
Leaf biomass	13	6.75	<.01	135	1.63	<.01	24	1.02	.44	13	1.53	.11	136	1.21	.10	24	3.43	<.01
Total vegetative biomass	13	6.33	<.01	135	1.54	<.01	24	0.93	.56	13	1.66	.08	136	1.22	.09	24	4.25	<.01
Root biomass										13	2.32	.01	136	1.13	.20	24	3.96	<.01
Total biomass										13	2.04	.02	136	1.20	.11	24	4.56	<.01
Reproductive traits																		
Number of inflorescences	13	4.91	<.01	135	1.58	<.01	24	0.81	.72	13	5.26	<.01	136	1.01	.46	24	2.63	<.01
Seed number	13	3.64	<.01	135	1.71	<.01	24	0.82	.71	13	5.26	<.01	136	1.01	.46	24	2.63	<.01
Seed biomass	13	6.00	<.01	135	1.50	<.01	24	0.91	.59	13	1.98	.03	136	0.95	.63	24	2.86	<.01
1000‐seed weight	13	2.02	.02	135	1.05	.36	24	0.83	.70	13	129.46	<.01	136	1.04	.39	24	0.89	.61
Physiological traits																		
Relative chlorophyll content	13	5.23	<.01	135	2.30	<.01	24	3.19	<.01	13	2.98	.01	28					
Net photosynthetic rate	13	0.95	.50	128	3.17	<.01	20	12.79	<.01	13	1.37	.18	129	1.03	.44	23	0.70	.84
Stomatal conductance	13	1.42	.16	128	0.65	.98	20	0.79	.72	13	1.00	.45	129	0.79	.90	23	0.69	.85
Intercellular CO_2_ concentration	13	0.82	.63	128	1.26	.15	20	0.69	.82	13	0.54	.90	129	0.82	.86	23	1.79	.02
Transpiration rate	13	1.15	.33	128	2.27	<.01	20	6.81	<.01	13	1.08	.38	129	1.20	.16	23	1.33	.17

*Note*: In the models, *S. canadensis* population identity was included as a fixed effect‐independent variable, while family identity and plots were included as random effect variables. Statistical significance of the factors was set at *p* < .05. Values of some traits were measured in the first or second year only.

**TABLE A4:** Results of a redundancy analysis showing loadings of phenotypic traits of *Solidago canadensis* from 14 populations on the first three RDA axes (RDA1‐RDA3).

Trait	RDA1	RDA2	RDA3
Plant height	−0.012	0.073	−0.003
Basal diameter	−0.0025	−0.0032	−0.0010
Leaf length	0.014	0.0007	0.032
Leaf width	−0.0189	0.0106	0.0126
L/W ratio	0.071	−0.026	0.009
SLA	0.126	−0.061	−0.005
Stem biomass	−0.245	−0.043	0.0008

*Note*: The traits were measured in the first year of the experiment.

**TABLE A5:** Results of a redundancy analysis showing loadings of phenotypic traits of *Solidago canadensis* from 14 populations on the first three RDA axes (RDA1–RDA3).

Trait	RDA1	RDA2	RDA3
Plant height	0.38	0.002	−0.0008
Basal diameter	0.025	0.001	−0.0001
Total biomass	0.175	−0.011	0.001
Number of inflorescences	−0.023	−0.002	−0.00013
Seed number	−0.106	−0.009	−0.0007
Seed biomass	0.030	−0.0059	−0.0018
1000‐seed weight	0.051	0.0016	0.0006

*Note*: The traits were measured in the second year of the experiment.
